# Correction: Identifying and Characterizing Nodes Important to Community Structure Using the Spectrum of the Graph

**DOI:** 10.1371/annotation/1935b388-2831-4fb1-b8f2-914ab91c1ddc

**Published:** 2011-12-13

**Authors:** Yang Wang, Zengru Di, Ying Fan

There is an error in the funding statement. The correct funding statement is, "This work is supported by the Natural Science Foundation of China (NSFC) under grants No. 61174150, No. 70771011, and No. 60974084, the Program for New Century Excellent Talents in University of Ministry of Education of China (No. NCET-09-0228), and fundamental research funds for the Central Universities of Beijing Normal University. The funders had no role in study design, data collection and analysis, decision to publish, or preparation of the manuscript."

